# Snapshot of methanogen sensitivity to temperature in Zoige wetland from Tibetan plateau

**DOI:** 10.3389/fmicb.2015.00131

**Published:** 2015-02-19

**Authors:** Li Fu, Tianze Song, Yahai Lu

**Affiliations:** ^1^College of Resources and Environmental Sciences, China Agricultural UniversityBeijing, China; ^2^School of Life Science, Fudan UniversityShanghai, China; ^3^College of Urban and Environmental Sciences, Peking UniversityBeijing, China

**Keywords:** methanogenesis, methanogens, temperature sensitivity, Zoige wetland, Tibetan plateau

## Abstract

Zoige wetland in Tibetan plateau represents a cold environment at high altitude where significant methane emission has been observed. However, it remains unknown how the production and emission of CH_4_ from Zoige wetland will respond to a warming climate. Here we investigated the temperature sensitivity of methanogen community in a Zoige wetland soil under the laboratory incubation conditions. One soil sample was collected and the temperature sensitivity of the methanogenic activity, the structure of methanogen community and the methanogenic pathways were determined. We found that the response of methanogenesis to temperature could be separated into two phases, a high sensitivity in the low temperature range and a modest sensitivity under mesophilic conditions, respectively. The aceticlastic methanogens *Methanosarcinaceae* were the main methanogens at low temperatures, while hydrogenotrophic *Methanobacteriales, Methanomicrobiales*, and *Methanocellales* were more abundant at higher temperatures. The total abundance of *mcrA* genes increased with temperature indicating that the growth of methanogens was stimulated. The growth of hydrogenotrophic methanogens, however, was faster than aceticlastic ones resulting in the shift of methanogen community. Determination of carbon isotopic signatures indicated that methanogenic pathway was also shifted from mainly aceticlastic methanogenesis to a mixture of hydrogenotrophic and aceticlastic methanogenesis with the increase of temperature. Collectively, the shift of temperature responses of methanogenesis was in accordance with the changes in methanogen composition and methanogenic pathway in this wetland sample. It appears that the aceticlastic methanogenesis dominating at low temperatures is more sensitive than the hydrogenotrophic one at higher temperatures.

## Introduction

The temperature sensitivity of soil microbial activity has gained increasing attention in recent decades (Davidson and Janssens, 2006). The global surface temperature is expected to increase by 3.7—4.8°C by the year 2100 (IPCC: Climate Change, 2014). Understanding the response of soil microbial activity is imperative to predict the feedback of global climate change. Low temperature has been a major constraint to the degradation of organic matter in high latitude and high altitude regions, where large amounts of readily degradable organic carbon have been stored (Davidson and Janssens, 2006; Liu et al., 2011). These regions, however, are warming rapidly. Temperature sensitivity of soil microbial activity is critical to the vulnerability of carbon stocks in these areas.

Temperature sensitivity of soil respiration has been estimated recently using meta-analysis and modeling. Based on the published data, Bond-Lamberty and Thomson (2010) obtained the mean temperature dependence of terrestrial respiration (Q_10_) of 1.5. Temperature sensitivity might be influenced by environmental and biological factors such as microbial community structure and substrate availability. Mahecha et al. (2010) derived the so-called intrinsic temperature dependences, and showed an almost universal Q_10_ value (1.4 ± 0.1) for ecosystems ranging from croplands to mixed forests and woody savanna. Similarly, Yvon-Durocher et al. (2012) reported that the sensitivity of ecosystem respiration to seasonal changes in temperature was statistically indiscernible across environments from aquatic to terrestrial ecosystems. The reconciled temperature sensitivity was also revealed for methanogenic systems from pure cultures to natural ecosystems (Yvon-Durocher et al., 2014). Thus, the meta-analysis and modeling indicated a remarkable consistency in temperature sensitivity of either terrestrial respiration or methanogenesis. This contrasts strikingly with the huge diversity of microbiota and their metabolisms contained in terrestrial ecosystems (Karhu et al., 2014).

Cold wetlands including boreal and alpine fens are among the most important biogenic sources of atmospheric methane (Conrad, 2009; Kirschke et al., 2013). To understand and predict the production and emission of CH_4_, numerous studies have been conducted in these areas to determine the spatiotemporal patterns of CH_4_ fluxes and the structure and function of methanogenic archaeal communities (e.g., Yrjala et al., 2011; Godin et al., 2012; Yang et al., 2014). The key factors controlling methanogenic activity have been identified that include temperature, water level, vegetation, surface topography, substrate availability, soil pH and depth (Galand et al., 2002, 2003, 2005; Høj et al., 2008; Juottonen et al., 2008; Tian et al., 2012a,b). Temperature stands out among the most important factors (Høj et al., 2008; Juottonen et al., 2008). Quantitative characterization of temperature sensitivity of methanogenic activity, however, is rare. Zoige wetland is an open fen located in Tibetan plateau with the average altitude of 3500 m, mean annual temperature of around 1°C and mean annual precipitation of 650 mm; it covers a total area of 6180 km^2^ (Chen et al., 2009a,b). The vegetation was dominated by *Carex muliensis* and *Eleocharis valleculosa* (Cai et al., 1965). Despite the cold climate, it has been estimated that the mean annual emission of CH_4_ amounts to 0.65–1.0 Tg, accounting for about one third of total emissions from natural wetlands in China (Chen et al., 2013a,b). Similar as in boreal fens and high arctic peats (Høj et al., 2008; Juottonen et al., 2008), temperature has been identified as the major factor controlling the seasonal and annual CH_4_ emissions from Zoige wetland (Chen et al., 2009b, 2013a). Investigation of methanogen community revealed the prevalence of psychrophilic aceticlastic and methylotrophic methanogens in Zoige wetland (Zhang et al., 2008b). For instance, a methanol-utilizing pure culture isolated from this wetland, *Methanolobus psychrophilus*, showed the optimum growth at 18°C, being active down to 0°C but stopped growth at 25°C (Zhang et al., 2008a). Apparently, the significant emission of CH_4_ is related to the existence of cold-adapted methanogens in this wetland. It, however, remains unknown how the methanogen community and methanogenesis in Zoige wetland will respond to a warming climate.

The investigations in rice field soils have shown that the methanogen community shifted from a mixture of aceticlastic and hydrogenotrophic methanogens under mesophilic conditions to the dominance of hydrogenotrophic *Methanocellales* at above 40°C (Fey and Conrad, 2000; Peng et al., 2008; Rui et al., 2011) or to the dominance of aceticlastic *Methanosaetaceae* and *Methanosarcinaceae* when temperature decreases to 15°C (Chin et al., 1999; Conrad et al., 2009). A preliminary study in Zoige wetland also showed the change in composition of methanogens and the pathway of methanogenesis between 15 and 30°C (Zhang et al., 2008a). We hypothesized that a shift in methanogenic community could induce change in temperature sensitivity of CH_4_ production in Zoige wetland. The purpose of the present study, therefore, was to determine the effects of temperature on methanogen composition and methanogenic activity and to evaluate if the temperature sensitivity of CH_4_ production in Zoige wetland was related with changes in methanogen community and methanogenic pathway. A laboratory incubation experiment was conducted with a soil sample collected from Zoige wetland. The incubations under different temperatures allowed us to explicitly link the sensitivity of methanogenic activity to the structure of methanogen community and the methanogenic pathways.

## Experimental methods

### Soil sampling and anaerobic incubation

The soil sample was collected from an open fen close to the Wetland National Nature Reserve of Zoige located in Qinghai-Tibetan Plateau (33°47′ N, 102°57′ E). The sampling site is covered dominantly by *Carex muliensis*. The soil is flooded periodically depending on precipitation and meltwater runoff. The microtopography consists of hummocks and hollows. The water table level was about 5 cm below the hollow surface at the time of sampling. Soil samples were collected on 25 July 2012 in about a square meter area at the depth of 5–20 cm below the hollow surface. Vegetation and organic debris was removed by hands during the sampling. About 10 kilograms of wet soil samples were placed in an ice box and transported to the laboratory within 24 h for immediate processing. The soil sample had the following characteristics: pH 7.5, organic C of 152.6 g kg^−1^, total N of 10.6 g kg^−1^, and C:N of 14.4. Soil slurries were prepared by mixing soil samples with autoclaved and degassed water. The slurries were passed through 2-mm sieves to homogenize and remove the coarse materials. Thirty grams of soil slurry was filled into 50-ml glass bottles with the final soil (d.w.) to water ratio of 1:3.5. The bottles were closed with butyl stoppers and flushed with N_2_. Soil slurries were incubated for 81 days at 10°C, 15°C, 20°C, 25°C, 30°C, and 35°C, respectively. Each temperature treatment was carried out in triplicate.

### Measurement of gases and volatile fatty acids

Gas samples (0.1 ml) were taken from headspace with a pressure-lock precision analytical syringe (Baton Rouge, LA, USA). The concentrations of CH_4_, CO_2_, and H_2_ were analyzed using gas chromatographs GC-7890 (Agilent Technologies, USA) equipped with a thermal conductivity detector. The ^13^C abundance (δ^13^C) of CH_4_ and CO_2_ was analyzed by a gas chromatography-isotope ratio mass spectrometry system (Yuan and Lu, 2009). Liquid samples (0.5 ml) were taken with sterile syringes and centrifuged 15 min at 17,949 × *g* at 4°C. The supernatant was collected, passed through 0.25-μm-pore-size filters, and stored at −20°C. Acetate and propionate were analyzed with an HPLC-1200 using a Zorbax SB-AQ C18 column (Agilent Technologies, USA).

Temperature sensitivity was calculated according to the Arrhenius equation:

(1) ln P=E(−1/RT)+M

where P is the rate of CH_4_ production, E is the activation energy (eV), T is the absolute temperature (K), R is the Boltzmann constant (8.623 × 10^−5^ eV K^−1^), and M is the theoretical rate of CH_4_ production in the absence of activation energy. The maximal rate of CH_4_ production was obtained from the cumulating curve of CH_4_ partial pressure in the headspace of incubation bottles. To incorporate enzyme concentration into the calculation, the maximal rates of CH_4_ production were normalized against the total abundances of *mcrA* that were quantified by real time PCR as described below.

### Nucleic acid extraction and purification

The total DNA of soil samples was extracted using the protocol by Ma et al. (2012). Briefly, 2 g of soil slurry was extracted sequentially with TPMS buffer (50 mM Tris-HCl [pH 7.0], 1.7% [wt/vol] polyvinylpyrrolidone K25, 20 mM MgCl_2_, 1%[wt/vol] sodium dodecyl sulfate) and phenol-based lysis buffer (5 mMTris-HCl [pH 7.0], 5 mM Na_2_ EDTA, 1% [wt/vol] sodium dodecyl sulfate, 6% [vol/vol] water-saturated phenol). Beads-beating was performed in FastPrep-24 (MP Biomedicals, USA). The supernatants were further extracted with water-saturated phenol, phenol-chloroform-isoamyl alcohol (25:24:1 [vol/vol/vol]), and chloroform-isoamyl alcohol (24:1 [vol/vol]). The extracts were purified by cold ethanol and sodium acetate. The quality and purity of DNA were checked by 1% agarose gel electrophoresis and NanoDrop1000 spectrophotometry (NanoDrop Technologies, Wilmington, DE).

### Terminal restriction fragment length polymorphism (T-RFLP) analysis

PCR amplification and terminal restriction fragment polymorphism (T-RFLP) analyses of archaeal 16S rRNA gene fragments followed the protocols described in Peng et al. (2008). PCR was carried out using the primer set Ar109f and Ar915r (Lueders et al., 2004). The 5′ end of the Ar915r primer was labeled with 6-carboxyfluorescein. The 50-μl reaction mixture contained 1 μl of DNA template (in 1:100 dilution of original extracts), 5 μl of 10 × buffer, 3 μl of 25 mM MgCl_2_, 1 μl of a 10 mM concentration of the deoxynucleoside triphosphates, 0.5 μl of each primer (50 μM), and 2.5 U of Tag DNA polymerase (TaKaRa). The thermal profile was as follows: 3 min at 94°C; 32 cycles of 60 s at 94°C, 45 s at 52°C, and 90 s at 72°C; and finally 5 min at 72°C. The PCR product was purified using an agarose gel DNA extraction kit (TaKaRa) and digested at 65°C for 3.5 h by *TaqI* (Fermentas, Canada). The digestion products were purified with SigmaSpin Post-Reaction Clean-Up Columns (Sigma), and a portion was mixed with deionized formamide and the internal standard GeneScan-1000 LIZ (Applied Biosystems). The mixtures were denatured for 3 min at 95°C, and the DNA fragments were size separated using a 3730 × l Genetic Analyzer (Applied Biosystems). The percent abundance (A*p*) of individual terminal restriction fragments (T-RFs) were calculated as the percentage of each peak height in the sum of all peak heights in a given T-RFLP profile and only those T-RFs with A *p* > 1% were considered in further analyses (Noll et al., 2005).

### Cloning, sequencing, and phylogenic analysis

Three clone libraries of the archaeal 16S rRNA genes were constructed from soil slurries incubated 49 days at 15°C, 25°C, and 35°C, respectively. The PCR amplification used the same primers as indicated above without FAM labeling. PCR products were purified and ligated into the pMD19-T vector (TaKaRa) according to the manufacturer's instructions. Plasmids were transformed into *E. coli* cells, and more than 100 clones were randomly selected from each clone library and sequenced with an ABI 3730 × l sequencer using BigDye Terminator cycle sequencing chemistry (Applied Biosystems) (Peng et al., 2008; Rui et al., 2009). Rarefaction curves (Figure S1) were calculated for three clone libraries according to the method described by Schloss and Handelsman (2005). Coverage of each clone library was calculated according to methods described by Good (1953) using the formula 1 − (n1/N)] × 100 (*n*1 and N denote the number of sole OTUs and total number of clones in a library, respectively). The coverage of analyzed clones by phylotype-richness estimates were 89, 92, and 81% for 15°C, 25°C, and 35°C treatment respectively, indicating that the clone libraries were adequately examined. Phylogenetic trees were constructed using the neighbor-joining algorithm according to the protocol of Lueders and Friedrich (2000), and bootstrap analysis implemented 1000 replicates.

### Quantitative (real-time) PCR analysis

Quantitative PCR of *mcrA* genes were carried out in a 7500 real-time PCR system (Applied Biosystems) using the primer pair mlas and *mcrA*-rev (Steinberg and Regan, 2008). Quantitative PCR was performed in a total volume of 25 μl containing 12.5 μl of Power SYBRsGreen PCR Master mix kit (Applied Biosystems), 0.75 μl (10 μM) primers, 200 ng bovine serum albumin and 2 μl of a 100:1 dilution of DNA sample. The thermal cycles and fluorescence signal acquisition followed the protocols described in (Steinberg and Regan, 2008). The DNA standards were prepared from the cloned sequence of *mcrA* gene, which were further amplified with vector-specific primers. The PCR products were purified with a UNIQ-10 column kit (Sangon Biotech, Shanghai, China). The obtained PCR products were quantified using the PicoGreen dsDNA quantification kit (Invitrogen, Eugene, OR) and then converted into the copy number of DNA molecules per unit volume ranging from 1.0 × 10^3^ to 1.0 × 10^8^ copies μl^−1^. Three replicates of each measurement were done.

### Nucleotide sequence accession numbers

The sequences of the 16S rRNA clones obtained in this study have been deposited in the EMBL nucleotide sequence database under the following accession numbers: KJ644784-KJ645071.

## Results

### Temperature sensitivity of methanogenesis

**Figure 1:**
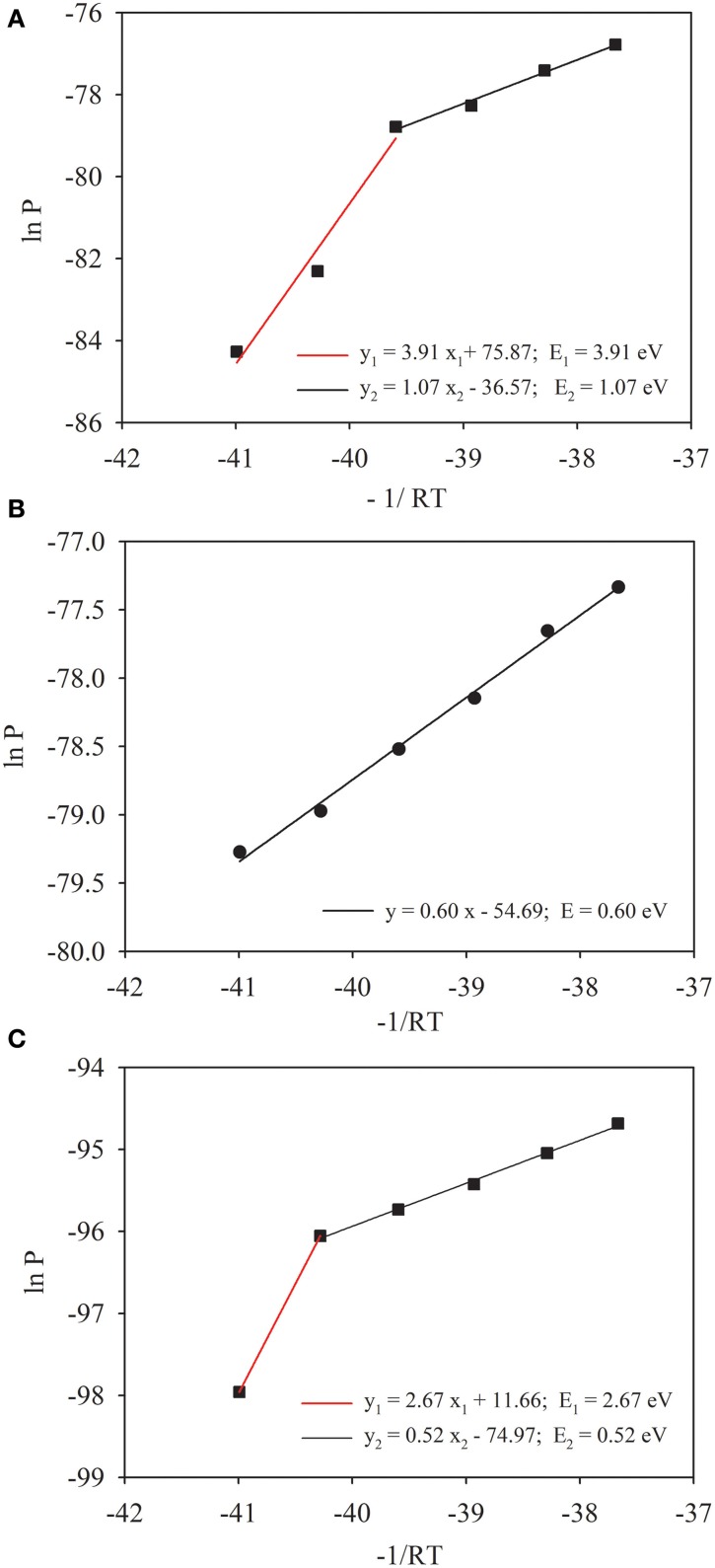
**Temperature dependences of CH_4_ (A) and CO_2_ (B) production in Zoige wetland**. Temperature dependence was characterized through plotting Arrhenius equation with “lnP” as a function of “−1/RT,” where P is the maximal rate of CH_4_ production, T is the absolute temperature and R is the Boltzmann constant (8.623 NU × 10^−5^ eV K^−1^). To take into account the effect of enzyme abundance, the maximal rate of CH_4_ production was normalized against the abundance of *mcrA* **(C)**. Solid lines [except the red line in **(C)**] are the regression fits, where the slope indicates the apparent activation energy E (eV).

Production of CH_4_ showed a lag phase that increased with the decrease of temperature (Figure S2). Emission of CO_2_ into the headspace, however, occurred immediately in all incubations. Accumulation of CH_4_ in the headspace increased markedly with the increase of temperature. We calculated the rate of CH_4_ production at each incubation temperature (Figure S3). The rate reached to maxima around 20 days at 30°C and 35°C, but delayed substantially at lower temperatures. The maximal rates corresponded roughly to substrate availability inferred from the dynamics of acetate and propionate in incubations (Figure S4). Based on maximal rates of CH_4_ production, we calculated the activation energy of methanogenesis according to Arrhenius equation (Figure 1). It revealed that the temperature sensitivity could be separated into two phases with the first activation energy of 1.07 eV between 20°C and 35°C and the second 3.91 eV at lower temperatures (Figure 1A). Similar calculation produced only a single value (0.60 eV) for production of CO_2_ across the temperature range tested (Figure 1B). Since temperature dependence could be influenced by enzyme concentration, we quantified *mcrA* (Table 1), the gene encoding the subunit A of methyl coenzyme-M reductase that metabolized the last step of CH_4_ production. The activation energy was recalculated using the maximal rates of CH_4_ production normalized against the maximal abundance of *mcrA*. The pattern of temperature dependences did not change, i.e., showing two phases of temperature sensitivity (Figure 1C). The values of activation energy, however, decreased to 0.52 eV in upper temperature range (15–35°C) and 2.67 eV in lower temperature range (<15°C).

**Table 1:** **Temperature and time dependent total *mcrA* gene abundances^a^**.

	**Days**	**Temperature (°C)**					
		**10**	**15**	**20**	**25**	**30**	**35**
*mcrA* (×10^6^ copies)	3	ND	ND	ND	ND	ND	ND
	24	ND	ND	ND	0.17 ± 0.02	7.61 ± 1.14	6.12 ± 1.15
	49	0.50 ± 0.04	0.46 ± 0.17	1.36 ± 0.21	0.52 ± 0.09	6.29 ± 1.62	7.50 ± 1.37
	65	0.49 ± 0.05	0.50 ± 0.12	3.50 ± 0.24	25.13 ± 1.66	45.52 ± 1.72	59.42 ± 9.30
	81	0.88 ± 0.24	0.93 ± 0.29	22.9 ± 2.72	28.31 ± 6.93	17.29 ± 2.86	55.37 ± 16.41

a *Values are means ± standard errors (n = 3); ND means not detected*.

### Community structure and methanogenic pathway

**Figure 2:**
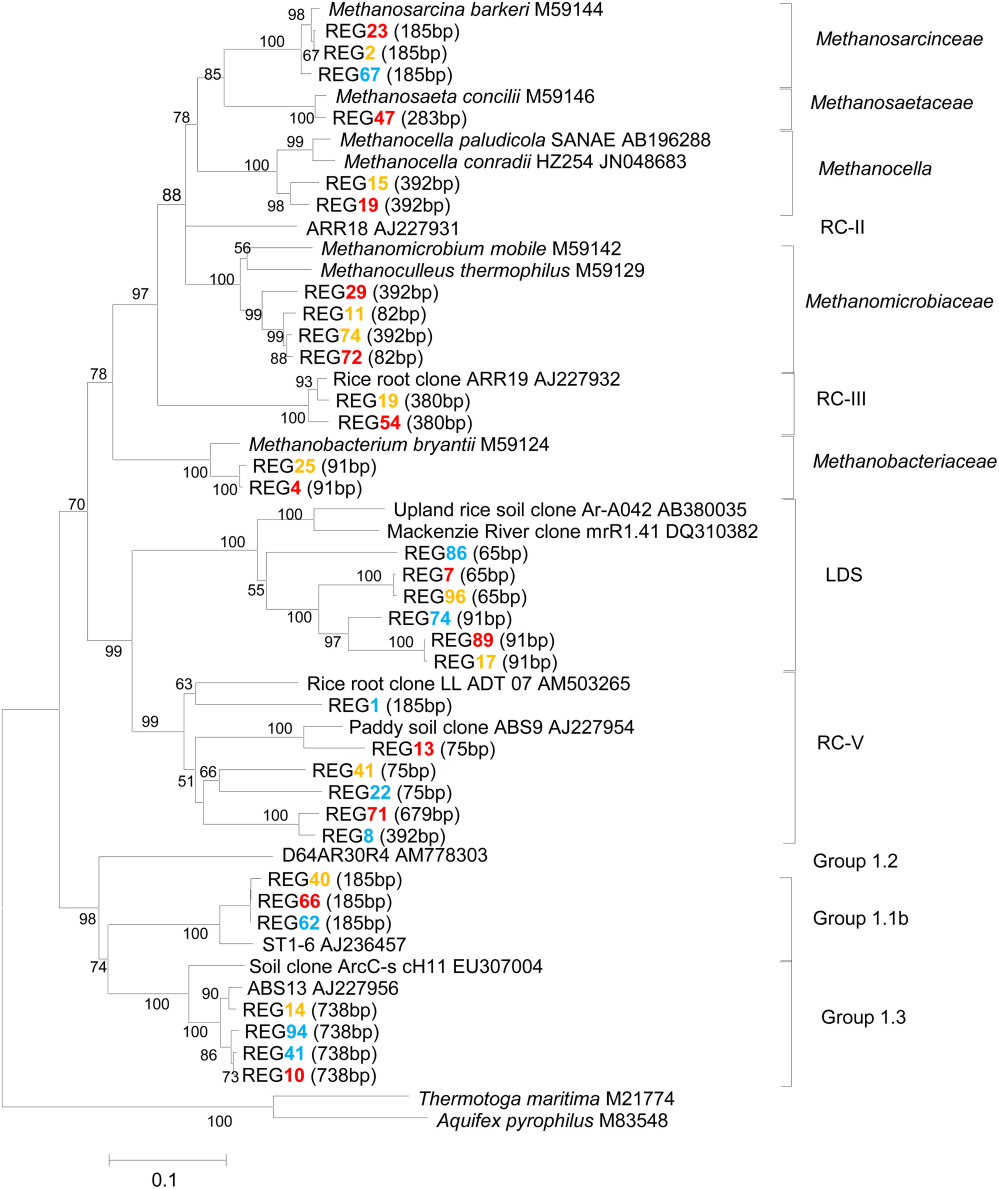
**Phylogenetic relationship of representative archaeal 16S rRNA gene clone sequences generated from our Zoige wetland samples incubated 49 days at 15°C (blue), 25°C (orange) and 35°C (red), respectively**. Clone libraries were constructed using primer set Ar109f and Ar915r, which produced about 800 bp size fragments. Sequences of this size were used for constructing the phylogenetic tree. The scale bar represents 10% sequence divergence. GenBank accession numbers of the reference sequences are indicated and *in silico* T-RF sizes are given in parentheses. Bootstrap values (%) were generated from 1000 replications and indicated at individual nodes.

Methanogen community was analyzed by using cloning, sequencing and T-RFLP analysis of the archaeal 16S rRNA genes. Three hundreds of 16S rRNA clones were retrieved from slurries incubated 49 days at 15°C, 25°C, and 35°C, respectively. The phylogenetic analysis of clone sequences showed that the archaeal community consisted of *Methanosarcinaceae, Methanosaetaceae, Methanocellales, Methanomicrobiaceae, Methanobacteriaceae*, and the uncultured euryarchaeotal RC-III, RC-V, LDS cluster and the crenarchaeotal group 1.1b and group 1.3 (Figure 2). The crenarchaeotal group 1.1b dominated accounting for over 40% of total archaeal clones (Figure S5). The relative abundances of methanogens and LDS cluster, however, increased relatively at 25°C and 35°C, while that of crenarchaeotal group 1.1b decreased.

**Figure 3:**
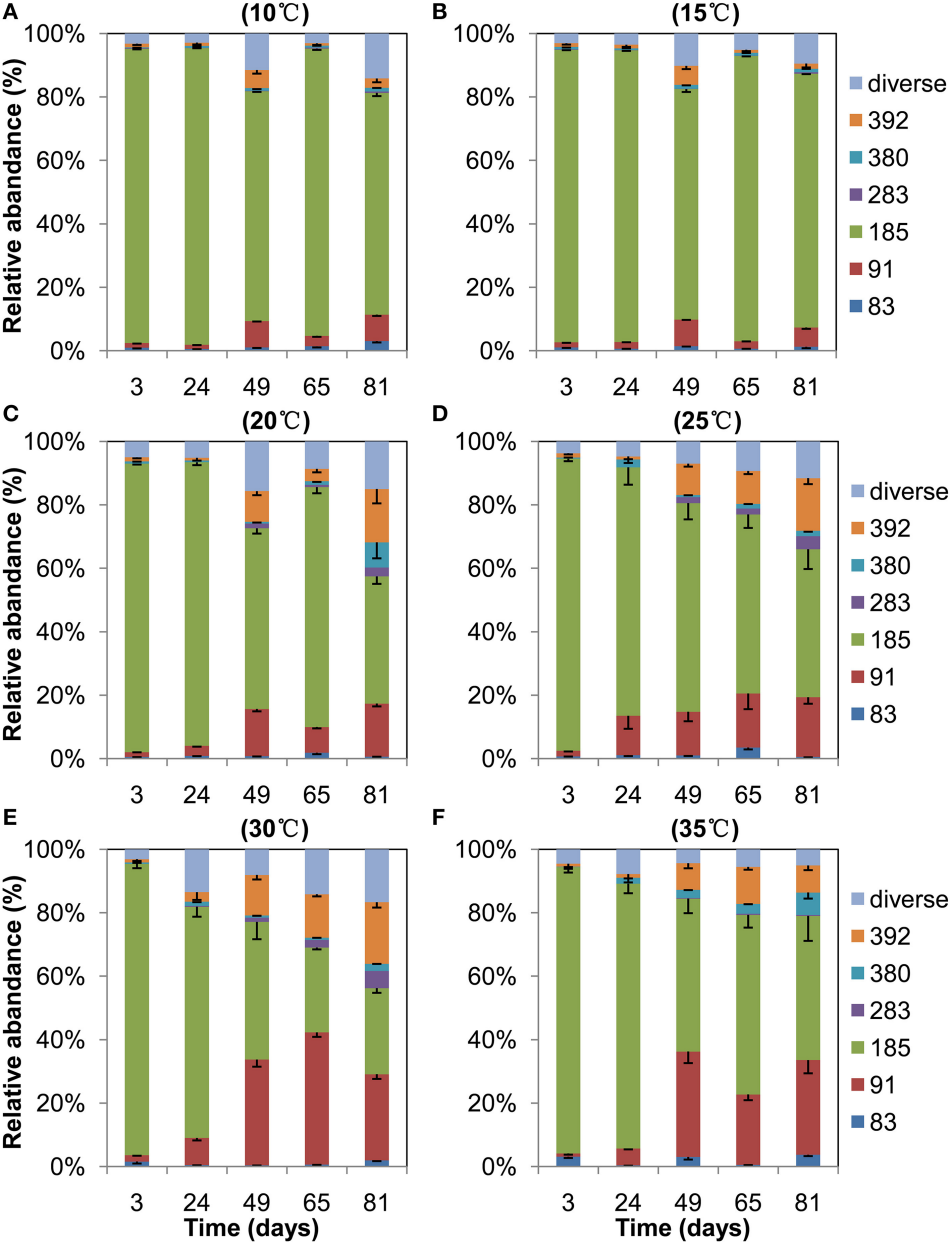
**T-RFLP profiles of the archaeal community in anaerobically incubated Zoige wetland soil at 10°C (A), 15°C (B), 20°C (C), 25°C (D), 30°C (E), and 35°C (F), respectively**. Data are means minus standard error (*n* = 3). Only major T-RFs are shown, the minor T-RFs are combined as Diverse.

T-RFLP profiles of archaeal 16S rRNA genes revealed that the 185-bp T-RF was predominant at the beginning and it remained dominant throughout the incubation at 10°C and 15°C (Figure 3). The relative abundance of this T-RF, however, decreased with incubation at higher temperatures. In contrast, the relative abundance of the 91-bp and 392-bp T-RFs increased over time in the incubations at 20°C and above. *In silico* analysis of clone sequences indicated that the 185-bp T-RF was related to *Methanosarcinaceae* and crenarchaeotal group 1.1b; the 91-bp T-RF to *Methanobacteriaceae* and LDS cluster, and the 392-bp T-RF to *Methanomicrobiaceae* and *Methanocellales* (Figure 2). Thus, the analyses of T-RFLP and clone sequences indicated that *Methanosarcinaceae* (185 bp) dominated the methanogen community at 10°C and 15°C, while the hydrogenotrophic methanogens *Methanobacteriales* (91 bp), *Methanomicrobiales* and *Methanocellales* (392 bp) increased when temperature increased to 20°C and above. The total abundance of *mcrA* increased with the increase of temperature (Table 1), indicating that the growth of methanogens was stimulated. Apparently, the growth of hydrogenotrophic methanogens was faster than aceticlastic ones, resulting in the shift of methanogen community.

**Figure 4:**
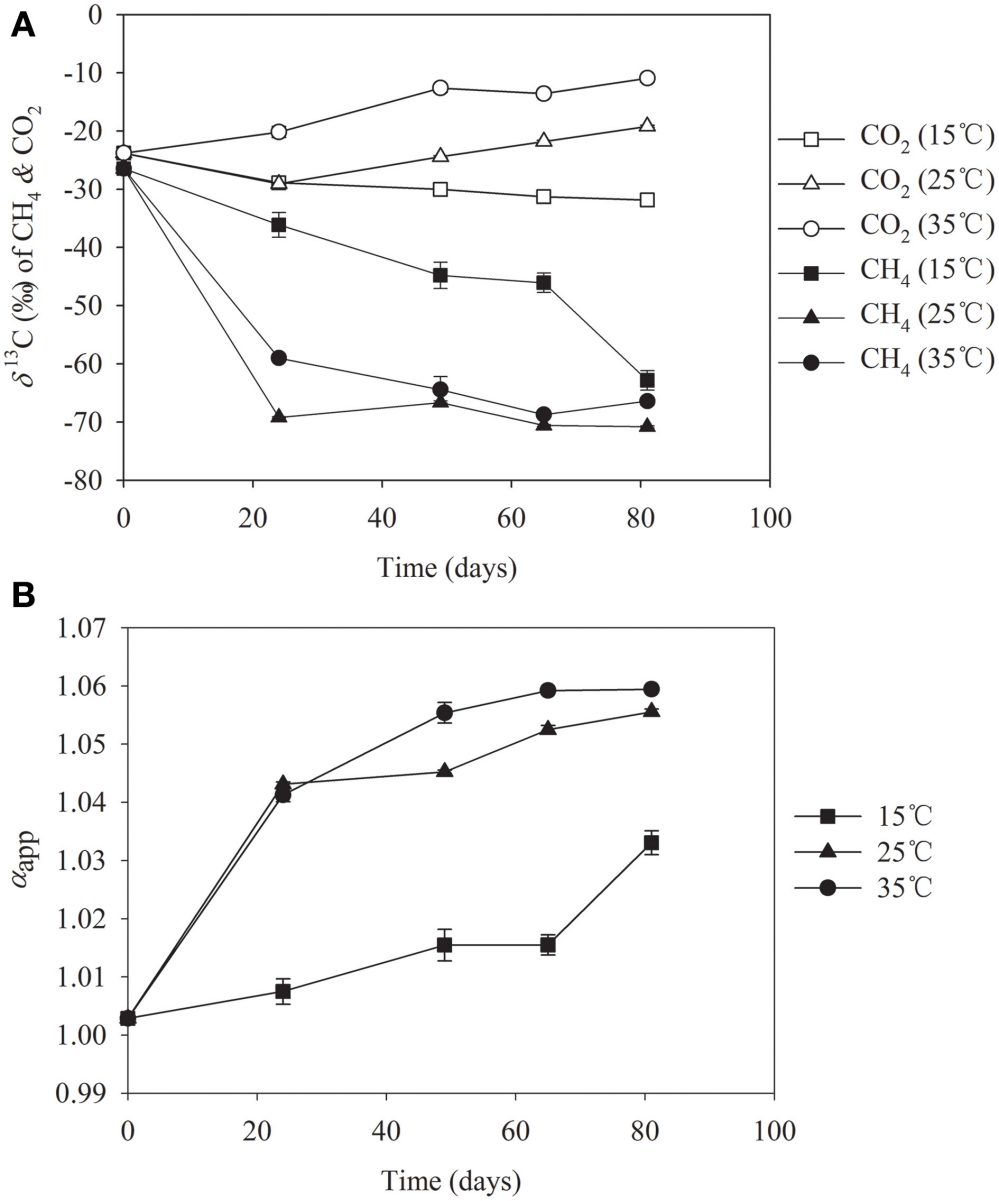
**δ^13^C values of CO_2_ and CH_4_ (A) and the apparent fractionation factor (α_app_) (B) in slurries of Zoige wetland incubated at 15°C (■), 25°C (▼), and 35°C (●), respectively**.

To determine if the pathway of CH_4_ production was altered by temperature, we analyzed the δ^13^C abundances of CH_4_ and CO_2_ in incubations at 15°C, 25°C and 35°C, respectively (Figure 4). It is known that CH_4_ produced from CO_2_ reduction is more depleted in δ^13^C compared with CH_4_ produced from acetate cleavage (Conrad et al., 2009). The relative contribution of hydrogenotrophic versus aceticlastic methanogenesis, thus, can be inferred from isotopic signatures. We found that the δ^13^C values of CH_4_ were between −60 and −70‰ at 25°C and 35°C after day 20 when CH_4_ production was most active, while the values at 15°C were much higher (Figure 4A). In correspondence, the δ^13^C value of CO_2_ increased slightly in incubations at 25°C and 35°C, but showed decreasing tendency at 15°C. The apparent isotopic fractionation factor [α_app_ = ^13^CO_2_ + 10^3^)/(δ ^13^CH_4_ + 10^3^)] showed a gradual increase from 1.04 to 1.06 in incubations at 25°C and 35°C, but remaining lower than 1.03 over the incubation at 15°C (Figure 4B). The α_app_ value of 1.04 is characteristic typically for CH_4_ production from both CO_2_ and acetate (Conrad et al., 2009). The decrease of δ^13^C values of CH_4_ and the increase of α_app_ at high temperatures indicate that more of CH_4_ was produced from CO_2_ reduction. The δ^13^C data, therefore, indicated that methanogenic pathway shifted from the aceticlastic methanogenesis at 15°C to a mixture of both hydrogenotrophic and aceticlastic methanogenesis at 25°C and 35°C. This shift was in coincidence with the change in methanogen community as described above.

## Discussion

We showed here that temperature sensitivity of CH_4_ production in a Tibetan wetland soil sample changed with temperature. Specifically, two phases of temperature dependence can be distinguished, a high sensitivity in the low temperature range (<15°C) and a modest sensitivity under mesophilic conditions. This change of temperature sensitivity was in accordance with the shifts of methanogen composition and methanogenic pathway. It appeared that the sensitivity of aceticlastic methanogenesis was greater than that of hydrogenotrophic methanogenesis. The temperature sensitivity of methanogenesis revealed in the present experiment was in contrast to the meta-analysis and modeling that showed a universal temperature dependence of methane production across different systems (Yvon-Durocher et al., 2014). It, however, was in line with the finding that the temperature sensitivity differed markedly between photosynthesis, respiration and methanogenesis (Macdonald et al., 1998; Van Hulzen et al., 1999; Hartley et al., 2006; Zou and Gao, 2013), which indicated the dependence of temperature sensitivity on metabolic mechanisms.

Temperature sensitivity was known to be influenced by substrate availability and enzyme concentration (Davidson and Janssens, 2006). We used the maximal rate of CH_4_ production to reduce the influence of substrate availability. The obtained sensitivity (1.07 eV) at the upper temperature range (20–35°C) was close to that derived from the meta-analysis (Yvon-Durocher et al., 2014). But when the enzyme factor (i.e., methanogen biomass) was incorporated, a much lower sensitivity (0.52 eV) was obtained. This difference was apparently due to the growth of methanogen populations with increasing temperature. The seasonal change (growth) in methanogen populations has been often observed in various ecosystems (He et al., 2014; Kanta Gaihre et al., 2014; Sabrekov et al., 2014; Wei et al., 2014). To obtain the intrinsic temperature dependence, the growth of methanogens should be taken into account.

In contrast to CH_4_ production, CO_2_ production showed a single sensitivity factor over the temperature range tested. This pattern and the activation energy (0.60 eV) were in agreement with previous predictions from the meta-analysis (Yvon-Durocher et al., 2012). One possibility might be that the metabolic mechanisms for CO_2_ production did not change with temperature. Cautions, however, have to be taken with this explanation, because the mechanisms for CO_2_ production in anoxic slurries are complicated and remain unclear, and in addition CO_2_ in the headspace is in equilibrium with liquid that is controlled by slurry pH. We did not measure slurry pH and hence the effect of chemical equilibrium was not counted.

We found a very high sensitivity of CH_4_ production in low temperature range. The methanogen community was dominated by *Methanosarcinaceae* at these temperatures. Members of *Methanosarcina* are substrate-versatile, using acetate, hydrogen and methylated C1 compounds for methanogenesis. The ^13^C signatures of CH_4_ and CO_2_, however, indicated that it was the aceticlastic pathway that dominated methanogenesis at low temperatures. At higher temperatures, the relative abundance of hydrogenotrophic methanogens, consisting of *Methanobacteriales, Methanomicrobiales* and *Methanocellales*, increased. We did not detect *Methanomicrobiales*-like Fen Cluster as observed often in acidic boreal fens (Galand et al., 2005; Juottonen et al., 2008). This was probably due to the neutral condition (pH 7.5) in our wetland soil sample. Despite different compositions, the shifting pattern of methanogen community and methanogenic pathway upon temperature changes is consistent with previous studies on sediment (Conrad, 1999; Glissmann et al., 2004), rice soil (Fey and Conrad, 2000; Peng et al., 2008) and high arctic peat (Høj et al., 2008). Thus, it appears that the shift of methanogen community and methanogenic pathway in response to temperature change is ubiquitous across ecosystems.

In seasonality studies under field conditions, other factors like vegetation growth, substrate availability and water level could also be important driving forces for the shift of methanogen community and methanogenesis (Juottonen et al., 2008). Temperature sensitivity factor of methanogenic activity has not been determined in previous studies. But in the study on boreal fen, it was shown that the relative abundance of *Methanosarcinaceae* increased while the hydrogenotrophic *Methanomicrobiales*-associated fen cluster (FC) was decreased in winter compared with summer (Juottonen et al., 2008). This shift of community composition was accompanied with a markedly enhanced temperature response for potential CH_4_ production in winter soil sample relative to summer sample. If similar pattern of temperature sensitivity as revealed in this study and that in boreal fen exists ubiquitously in cold environments, large increase of global CH_4_ emissions may eventually occur in a warming climate.

In summary, we showed that temperature responses of CH_4_ production in a Zoige wetland soil sample displayed a high sensitivity in the low temperature range and a modest sensitivity under mesophilic conditions. This change in sensitivity was correlated with shifts of methanogen community and methanogenic pathway. We have to indicate that only one soil sample was tested in the present study, hence representing only a snapshot of temperature sensitivity of methanogens in Zoige wetland. Further researches shall be necessary to elucidate the mechanisms of this pathway-dependent temperature sensitivity across different ecosystems and take it into account in the future modeling and prediction of climate change impacts and feedbacks.

### Conflict of interest statement

The authors declare that the research was conducted in the absence of any commercial or financial relationships that could be construed as a potential conflict of interest.
