# Supplementary material for: Snapshot of methanogen sensitivity to temperature in Zoige wetland from Tibetan plateau
Source: Front Microbiol. 2015 Feb 19;6:131. doi: 10.3389/fmicb.2015.00131 (PMC4333864; doi:10.3389/fmicb.2015.00131)
Supplement: Supplementary file 1 [file Image1.PDF]

Supplementary Material

Snapshot of methanogen sensitivity to temperature in Zoige wetland from Tibetan plateau

Li Fu [1] and Yahai Lu[1,2]*

[1]College of Resources and Environmental Sciences, China Agricultural University, Beijing 100193, China

[2]College of Urban and Environmental Sciences, Peking University, Beijing 100871, China

***Corresponding author:**

Yahai Lu
College of Urban and Environmental Sciences,
Peking University,
Beijing 100871, China.
Phone/Fax: 0086 10 62755683
E-mail: luyh@pku.edu.cn

1. Suplementary Figures

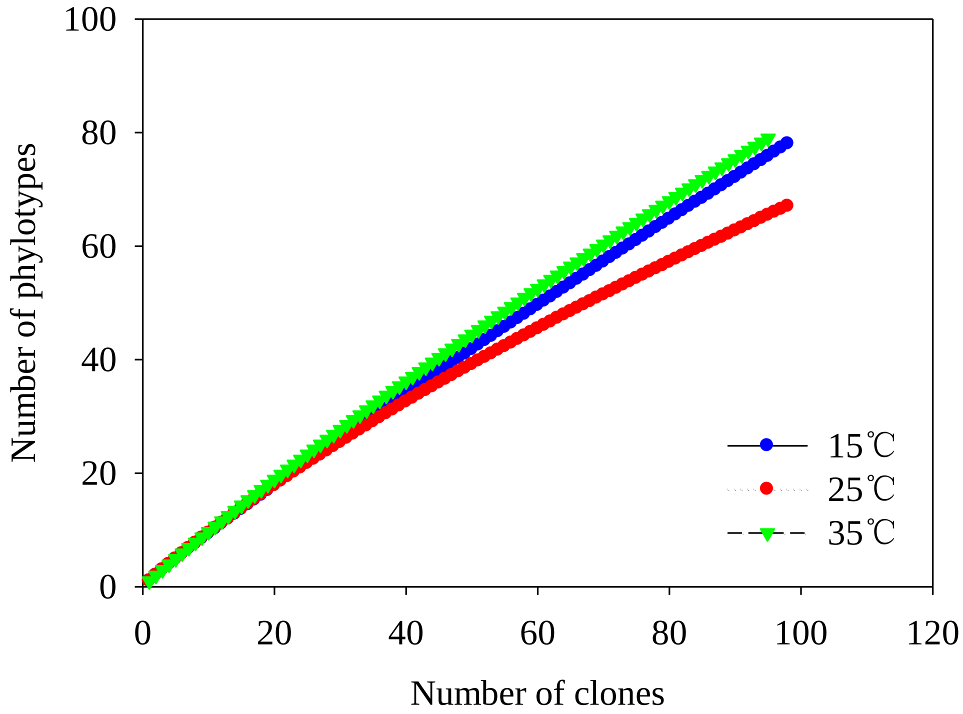

Supplementary Figure 1. Rarefaction curves constructed from three clone libraries of archaeal 16S rRNA gene.

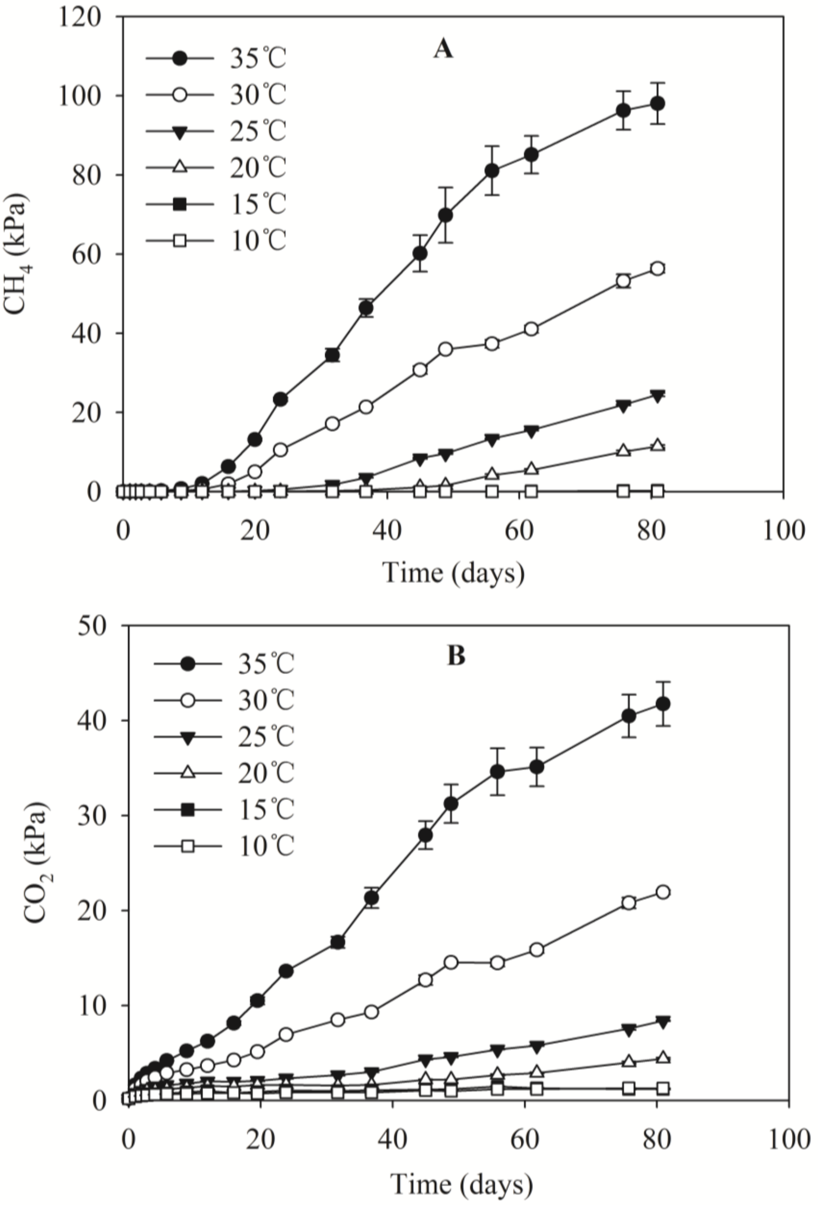

Supplementary Figure 2. CH_4 (A) and CO_2 (B) accumulated in slurries of Zoige wetland soil incubated at 10°C (□), 15°C (■), 20°C (△), 25°C (▼), 30°C (○) and 35°C (●). Data are means ± standard errors (n = 3).

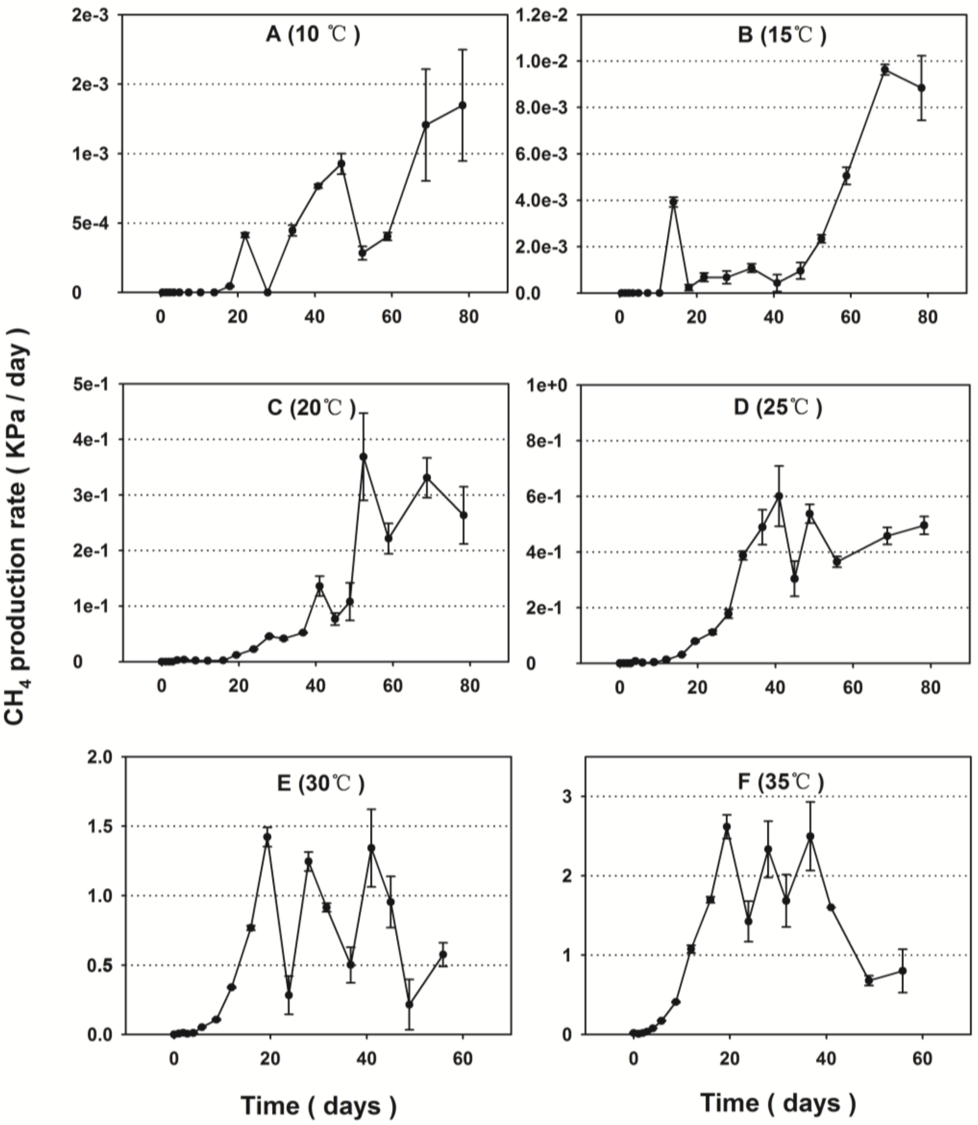

Supplementary Figure 3. Rates of CH_4 production calculated from the cumulative curves in illustrated in Figure S1. Data are means ± standard errors (n = 3).

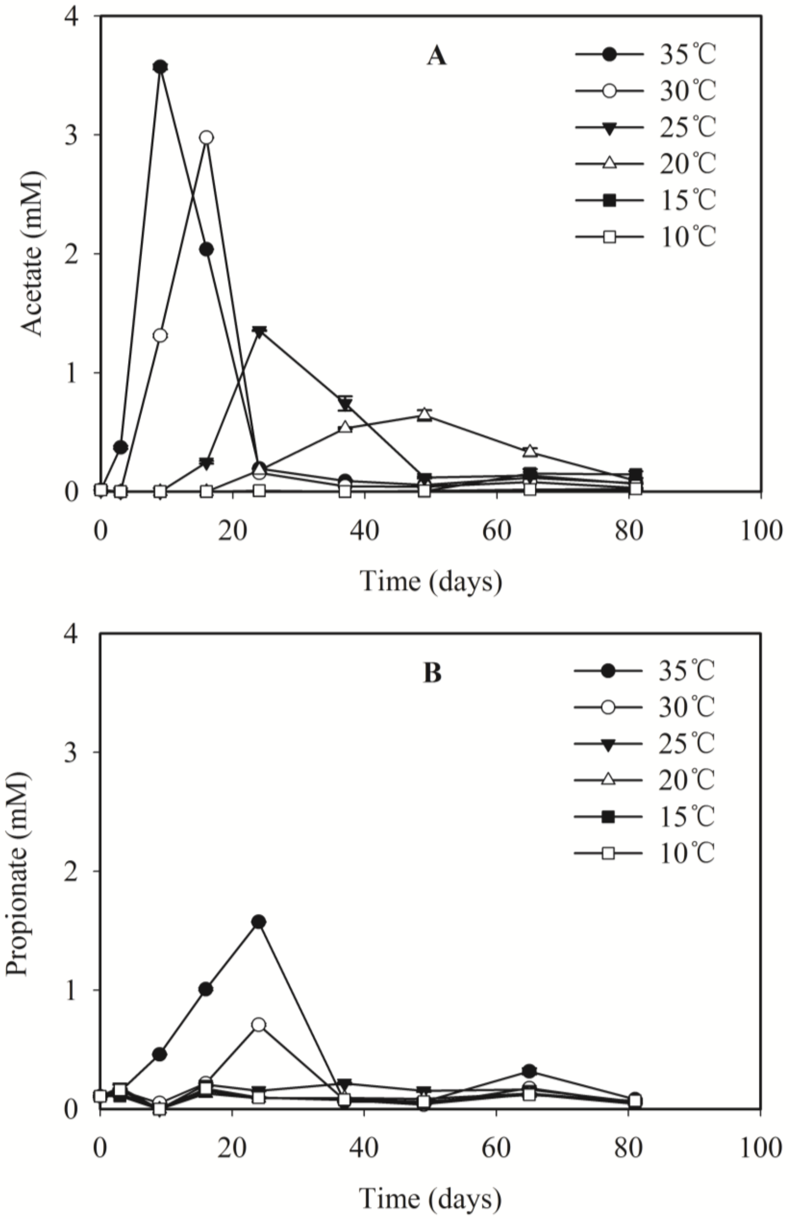

Supplementary Figure 4. Accumulation of acetate (A) and propionate (B) in slurries of Zoige wetland soil incubated at 10°C (□), 15°C (■), 20°C (△), 25°C (▼), 30°C (○) and 35°C (●). Data are means ± standard errors (n = 3).

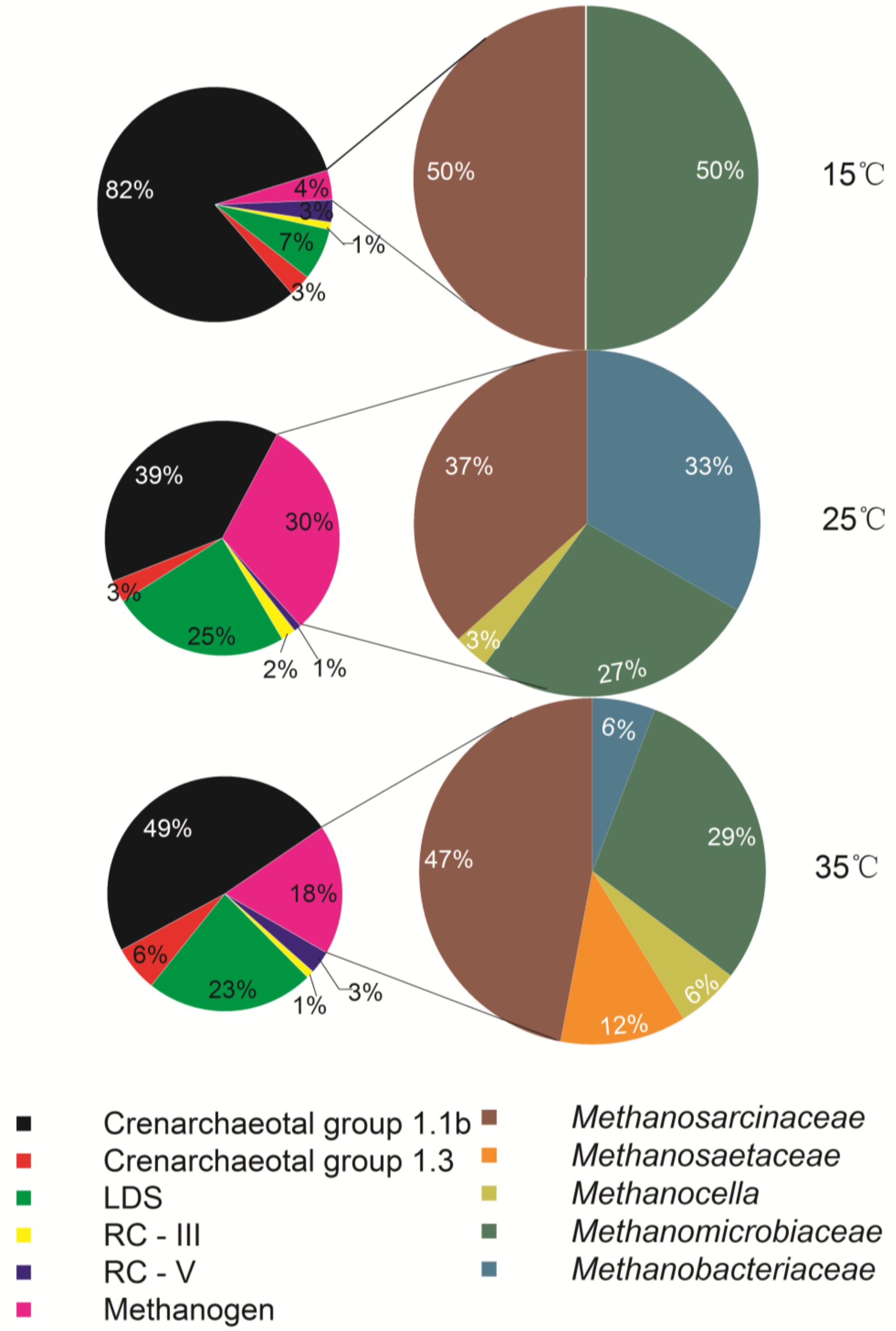

Supplementary Figure 5. Composition of 16S rRNA clone libraries derived from Zoige wetland slurries incubated 49 days at 15 ℃, 25 ℃ and 35 ℃, respectively. The community structure is represented by the relative abundance of clone sequences belonging to different phylogenetic clusters.
